# A new approach for approximating the p-value of a class of bivariate sign tests

**DOI:** 10.1038/s41598-023-45975-7

**Published:** 2023-11-05

**Authors:** Ibrahim A. A. Shanan, Ehab F. Abd-Elfattah, Abd El-Raheem M. Abd El-Raheem

**Affiliations:** 1 Department of Mathematics, Faculty of Education, Ain Shams University, Cairo, Egypt; 2 Department of Information Technologies, Management Technical College, Southern Technical University, Basra, Iraq

**Keywords:** Computational biology and bioinformatics, Mathematics and computing, Applied mathematics

## Abstract

Bivariate data are frequently encountered in many applied fields, including econometrics, engineering, physiology, biology, and medicine. For bivariate analysis, a wide range of non-parametric and parametric techniques can be applied. There are fewer requirements needed for non-parametric procedures than for parametric ones. In this paper, the saddlepoint approximation method is used to approximate the exact p-values of some non-parametric bivariate tests. The saddlepoint approximation is an approximation method used to approximate the mass or density function and the cumulative distribution function of a random variable based on its moment generating function. The saddlepoint approximation method is proposed in this article as an alternative to the asymptotic normal approximation. A comparison between the proposed method and the normal asymptotic approximation method is performed by conducting Monte Carlo simulation study and analyzing three numerical examples representing bivariate real data sets. In general, the results of the simulation study show the superiority of the proposed method over the asymptotic normal approximation method.

## Introduction

Recently, several experiments have been performed based on bivariate data. As a result, bivariate data analysis is critical in statistical research and is typical of many studies. One of the important problems associated with bivariate data is the problem of testing the symmetry of the two bivariate distributions. Suppose the observed bivariate data takes the form $Z=\left\{\left({x}_{1},{y}_{1}\right), \dots ,\left({x}_{n},{y}_{n}\right)\right\}$ where $\left({x}_{1},{y}_{1}\right), \dots ,\left({x}_{n},{y}_{n}\right)$ are assumed to be mutually independent from a bivariate population follow the F distribution. The problem is to test the symmetrical hypothesis.

$${H}_{0}:F\left(x,y\right)=F\left(y,x\right) \quad for \,all \left(x,y\right).$$

Sign testing is a common method of testing symmetry. Many statisticians and those interested in statistical inference have made many generalizations to the univariate sign test in order to obtain the corresponding test in the bivariate case. Work began on this point by both Hodges^1^ and Blumen^2^. After that, many studies appeared with the aim of providing and developing the sign tests for the bivariate case. In this regard, we can point out the contributions of Chatterjee^3^, Kohnen^4^, Dietz^5^, Brown and Hettmansperger^6^, Oja and Nyblom^7^, and Brown and Hettmansperger^8^. Brown et al.^9^ discussed the concepts of bivariate sign test and bivariate medians. Larocque et al.^10^ introduced an affine-invariant modification of the Wilcoxon signed-rank test for bivariate location problems. The advantage of this test over Jan and Randles^11^ test is that its asymptotic null distribution holds without assuming elliptical symmetry. Samawi^12^ introduced a bivariate sign test for the one-sample bivariate location problem using a bivariate ranked set sample. Ghute and Shirke^13^ developed a nonparametric control chart for monitoring the changes in the location of a bivariate process, the proposed chart is based on Bennett’s^14^ bivariate sign test.

The p-value plays an important role in hypothesis tests because of its important role in determining the acceptance or rejection of the null hypothesis. Therefore, approximating the exact p-value with high accuracy is a challenge in many statistical tests. In this context, the saddlepoint approximation method is suggested to approximate the exact p-value of a class of bivariate tests which takes the general linear form

1 $${\varvec{B}}=\sum_{i=1}^{n}{\beta }_{i}{{\varvec{U}}}_{i}+{\varvec{C}},$$

where ${{\varvec{U}}}_{i}$ is the vector of score function based on observation of the sample, ${\beta }_{i}$ is the vector of indicators which has a sequence of ones and zeroes, and ${\varvec{C}}$ is a constant vector possibly depending on observation of the sample.

The saddlepoint approximation method is basically just a method for approximating the density function. Daniels^15^ was the first one who initially proposed the general application of the saddlepoint approximation for density function. The cumulative distribution function in the univariate case was approximated by Lugannani and Rice^16^ depending on the proposal of Daniels. Skovgaard^17^ provided a double saddlepoint approximation for the conditional distributions. A saddlepoint approximation for a bivariate distribution function was introduced by Wang^18^. Abd-Elfattah^19^ introduced an accurate and easy approximation for the distribution function of bivariate class of random sum distributions using saddlepoint approximation technique. Abd-Elfattah^20^ approximated the exact permutation distribution of a class of two-sample bivariate tests using saddlepoint approximation technique. Abd-Elfattah^21^ used the saddlepoint approximation to approximate the distribution function of the bivariate symmetry test statistic under competing risk data. Abd El-raheem and Abd-Elfattah^22,23^ approximated the exact permutation distribution of a class of two-sample tests for cluster data under two different randomization designs. For more recent articles in the saddlepoint approximation method; see Kamal et al.^24,25^. In the end, we can mention a number of important and basic references on the subject of saddlepoint approximations, which highlight the importance and applications of saddlepoint approximations in many branches and fields of statistics, namely: Booth and Butler^26^, Strawderman^27^, Butler^28^, Abd-Elfattah and Butler^29^, Kwok and Zheng^30^.

As mentioned earlier, our goal is to approximate the mid-p value of a class of bivariate sign tests. The focus here is on the mid p-value rather than ordinary p-value since the ordinary p-value is too conservative in comparison to the mid p-value, see Abd-Elfattah^20^. Such a class of bivariate sign tests is presented in detail in “Bivariate sign tests” section. The bivariate saddlepoint approximation is applied to approximate the mid-p value of a class of bivariate sign tests in “Bivariate saddlepoint approximations” section. “Illustrative examples and simulation studies” section compares the performance of the saddlepoint approximation and the asymptotic normal method using numerical examples and simulation studies.

## Bivariate sign tests

This section presents two of the most frequently used bivariate sign tests in one sample problem. After that, we formulate the two statistics of such two tests in a general linear form to facilitate obtaining highly accurate approximation of exact p-value of such bivariate sign tests using bivariate saddlepint approximation method.

### Bivariate sign test of Blumen^2^

The bivariate sign test was provided by Blumen^2^ to test the hypothesis that the medians of two variables have a specific value. This test was created to be independent of correlation between the two variables. Let $\left({x}_{i},{y}_{i}\right)$ represent the bivariate sample points. In order to perform Blumen’s bivariate sign test, consider the $n$ axes created by drawing a line across each $\left({x}_{i},{y}_{i}\right)$ and the origin, and number the axes corresponding to the angle counterclockwise from the positive end of the horizontal axis. Let ${\gamma }_{i}=+ 1\, or\ - 1$ if the data point associated with the *i*th axis is higher (lower) than the horizontal axis. The center of gravity is calculated by computing the values at the intersection of the standardized vectors and the unit circle. Blumen’s test statistic is given by

2 $${l}_{1}=\sum_{i=0}^{n-1}{\gamma }_{i}\mathrm{cos}\left(\frac{\pi i}{n}\right) \text{ and } {l}_{2}=\sum_{i=0}^{n-1}{\gamma }_{i}\mathrm{sin}\left(\frac{\pi i}{n}\right).$$

If the null hypothesis is true, then ${l}_{1}$ and ${l}_{2}$ are approximately independent normal variables with mean zero and variance $n/2.$

Let ${\alpha }_{i}=\frac{{\gamma }_{i}+1}{2}$, then ${l}_{1}$ and ${l}_{2}$ in Eq. (2) become

$${l}_{1}= \sum_{i=0}^{n-1}2{\alpha }_{i} \cos\left(\frac{\pi i}{n}\right)-\sum_{i=0}^{n-1}\mathrm{cos}\left(\frac{\pi i}{n}\right),$$

and

$${l}_{2} = \sum_{i=0}^{n-1}2{\alpha }_{i} \sin\left(\frac{\pi i}{n}\right)-\sum_{i=0}^{n-1}\mathrm{sin}\left(\frac{\pi i}{n}\right),$$

where ${\alpha }_{i}=\left\{\mathrm{0,1}\right\}$. Now, the statistics ${l}_{1}$ and ${l}_{2}$ can be rewritten in the bivariate sign statistic form as

3 $${\varvec{L}}=\sum_{i=1}^{n}2{\alpha }_{i}\left(\begin{array}{c}\mathrm{cos}\left(\frac{\pi (i-1)}{n}\right)\\ \mathrm{sin}\left(\frac{\pi (i-1)}{n}\right)\end{array}\right)-\sum_{i=1}^{n}\left(\begin{array}{c}\mathrm{cos}\left(\frac{\pi \left(i-1\right)}{n}\right)\\ \mathrm{sin}\left(\frac{\pi \left(i-1\right)}{n}\right)\end{array}\right),$$

or in the form (1) with ${\beta }_{i}={\alpha }_{i}$ , ${{\varvec{U}}}_{i}= {2\left(\mathrm{cos}\left(\frac{\pi (i-1)}{n}\right),\mathrm{sin}\left(\frac{\pi (i-1)}{n}\right) \right)}^{T}$ and ${\varvec{C}}=-{\sum }_{i=1}^{n}{\left(\mathrm{cos}\left(\frac{\pi (i-1)}{n}\right),\mathrm{sin}\left(\frac{\pi (i-1)}{n}\right) \right)}^{T}.$

### Bivariate sign test of Brown et al.^9^

Brown et al.^9^ introduced another idea for bivariate symmetry test. Let ${{\varvec{z}}}_{1},\dots ,{{\varvec{z}}}_{n}$ be a sample drawn at random from a bivariate distribution. Brown et al.^9^ meant by symmetry here that ${{\varvec{z}}}_{{\varvec{i}}}-{\varvec{\mu}}$ and ${\varvec{\mu}}-{{\varvec{z}}}_{{\varvec{i}}}$ are identically distributed, where ${\varvec{\mu}}$ is the symmetry center. Thus, the null hypothesis of bivariate symmetry is defined by

$${\mathrm{H}}_{0}:{\varvec{\mu}}= 0.$$

The observed data can be represented in the following form

$${{\varvec{z}}}_{i}={\gamma }_{i}{{\varvec{x}}}_{i}={{\gamma }_{i}r}_{i}\left(\begin{array}{c}\mathrm{cos}({\varphi }_{i})\\ \mathrm{sin}({\varphi }_{i})\end{array}\right),$$

where ${\gamma }_{i}=1$ or $-1$ if ${{\varvec{z}}}_{i}$ is above or below the horizontal axis, respectively, ${r}_{i}$ is the *i*th radius, and $0\le {\varphi }_{1}\le {\varphi }_{2}\le \dots \le {\varphi }_{n}\le \pi$ are the ordered angles.

Under the null hypothesis, $P\left({\gamma }_{i}=1\right)=P\left({\gamma }_{i}=-1\right)=\frac{1}{2}$.

Let ${{\varvec{z}}}^{T}=\left({z}_{1},{z}_{2}\right)$, and ${\dot{{\varvec{z}}}}^{T}=\left(-{z}_{2},{z}_{1}\right)$, then the gradient vector at the origin (divided by n) is given by

$${\varvec{q}}=\frac{1}{2n}\sum_{i=1}^{n-1}\sum_{j=i+1}^{n}sign\left(\left|\begin{array}{cc}{z}_{i1}& {z}_{j1}\\ {z}_{i2}& {z}_{j2}\end{array}\right|\right)({\dot{{\varvec{z}}}}_{j}-{\dot{{\varvec{z}}}}_{i})=\frac{1}{2}\sum_{i=1}^{n-1}\sum_{l=i+1}^{n}{\gamma }_{i}{\gamma }_{l}({\gamma }_{l}{\dot{{\varvec{x}}}}_{l}-{\gamma }_{i}{\dot{{\varvec{x}}}}_{i}).$$

The statistic ${\varvec{q}}$ becomes simpler after some simplification as following:

4 $${\varvec{q}}=\frac{1}{2}\sum_{i=1}^{n}{\gamma }_{i}{{\varvec{w}}}_{i},$$

where ${{\varvec{w}}}^{T}=\left({w}_{1},{w}_{2}\right)$ and using ${{\varvec{x}}}_{n+i}=-{{\varvec{x}}}_{i}$ such that:

$${{\varvec{w}}}_{i}=\frac{1}{n}\sum_{l=1}^{n-1}{\dot{{\varvec{x}}}}_{i+l} .$$

The statistic ${\varvec{q}}$ is asymptotic normal with mean ${\varvec{\mu}}=E\left({\varvec{q}}|{H}_{0}\right)=0$ and covariance matrix ${\varvec{\sigma}}=\frac{1}{4}\sum_{i=1}^{n}{{\varvec{w}}}_{i}{{\varvec{w}}}_{i}^{T}$.

Let ${\alpha }_{i}=\frac{{\gamma }_{i}+1}{2}$ then ${\alpha }_{i}=\left\{\mathrm{0,1}\right\}$ and the statistic ${\varvec{q}}$ becomes

5 $${\varvec{q}}=\sum_{i=1}^{n}{\alpha }_{i}{{\varvec{w}}}_{i}-\frac{1}{2}\sum_{i=1}^{n}{{\varvec{w}}}_{i}.$$

It is clear that the statistic ${\varvec{q}}$ takes the same form of the linear statistic in Eq. (1) with ${\beta }_{i}={\alpha }_{i}$, ${{\varvec{U}}}_{i}={{\varvec{w}}}_{i}$ and ${\varvec{C}}=-\frac{1}{2}{\sum }_{i=1}^{n}{{\varvec{w}}}_{i}$.

## Bivariate saddlepoint approximations

The permutation distribution of the general from of the bivariate sign statistic in Eq. (1) is ${2}^{n}$. This distribution can be derived from the set $\{{\beta }_{1}, . . .,{\beta }_{n}\}$ of independent and identically Bernoulli (1/2) random variables. The bivariate sign statistic in (1) can be written as two sign statistics as

$${b}_{1}=\sum_{i=1}^{n}{\beta }_{i}{U}_{1i}+{c}_{1}\;\;\mathrm{and}\;\; {b}_{2}=\sum_{i=1}^{n}{\beta }_{i}{U}_{2i}+{c}_{2},$$

with $${{\varvec{U}}}_{i}={\left({U}_{1i},{U}_{2i}\right)}^{T}\, \mathrm{and}\, {\varvec{C}}={({c}_{1},{c}_{2})}^{T}.$$

Let ${{\varvec{B}}}_{0}=(\tau ,\upsilon )$ be observed value of ${\varvec{B}}$, it is possible to calculate the mid-p value of the statistic ${\varvec{B}}$ at ${{\varvec{B}}}_{0}$ as

6 $$\mathrm{mid}-\mathrm{p}\left({{\varvec{B}}}_{0}\right)=\mathrm{Pr}\left({b}_{1}> \tau ,{b}_{2}> \upsilon \right)+\frac{1}{2}\mathrm{Pr}\left({b}_{1}=\tau , {b}_{2}=\upsilon \right).$$

The $\mathrm{mid}-\mathrm{p}\left({{\varvec{B}}}_{0}\right)$ can be approximated using saddlepoint approximation of the bivariate CDF which was developed by Wang^18^. The approximate formula presented by Wang^18^ is an approximation of the bivariate cumulative distribution function as a generalization of the approximation presented by Lugannani and Rice^16^ which is the approximation of the univariate cumulative distribution function. Both approximations are an approximation of the intractable integrals resulting from calculating different forms of probabilities. These approximations totally depend on the cumulant generating function (CGF).

The joint CGF of ${b}_{1}$ and ${b}_{2}$ is given by

$$K\left(t,u\right)=\sum_{i=1}^{n}\mathrm{log}\left\{\frac{1}{2}+\frac{1}{2}{\text{exp}}\left(t{U}_{1i}+{uU}_{2i}\right)\right\}+\left(t{c}_{1}+u{c}_{2}\right).$$

Since ${{\varvec{B}}}_{0}=(\tau ,\upsilon )$ is the observed value of the statistic ${\varvec{B}}$, assume for fixed $(\tau ,\upsilon )$ that there exists a unique solution $\left({t}_{0},{u}_{0}\right)$ of the following equation

7 $$\left(\frac{\partial }{\partial t}K\left(t,u\right){\left.\right|}_{\left({t}_{0},{u}_{0}\right)},\frac{\partial }{\partial u}K\left(t,u\right){\left.\right|}_{\left({t}_{0},{u}_{0}\right)}\right)=\left(\tau ,\upsilon \right),$$

and $t={\widehat{t}}_{0}$ solves the equation

8 $$\frac{\partial }{\partial t}{K}_{1}\left(t\right)=\tau ,$$

where ${K}_{1}(t)$ is the CGF of ${b}_{1}$, and similarly it can be assumed that ${K}_{2}(u)$ is the CGF of ${b}_{2}$.

According to the saddlepoint approximation of the bivariate CDF, the $\mathrm{mid}-\mathrm{p}\left({{\varvec{B}}}_{0}\right)$ in (6) can be approximated as

9 $$\mathrm{mid}-\mathrm{p}\left({{\varvec{B}}}_{0}\right)\approx 1- \left({I}_{11}+{I}_{12}+{I}_{21}+{I}_{22}\right),$$

where $${I}_{11}\sim\Phi ({\tau }_{1},{\upsilon }_{1},{\rho }_{1})$$, $${I}_{12}\sim\Phi ({w}_{{u}_{0}})\phi ({\upsilon }_{0})\left\{{\upsilon }_{0}^{-1}-{({u}_{0}G)}^{-1}\right\}$$, $${I}_{21}=\Phi \left({\upsilon }_{0}\right)\phi \left({\tau }_{1}\right)\left\{{w}_{{u}_{0}}^{-1}-{t}_{0}^{-1}{[{K}_{tt}\left({t}_{0},{u}_{0}\right)]}^{-1/2}\right\},$$

$${I}_{22}=\mathrm{exp}\left\{K\left({t}_{0},{u}_{0}\right)-{t}_{0}\tau -{u}_{0}\upsilon \right\}\left\{{w}_{{u}_{0}}^{-1}-{t}_{0}^{-1}{[{K}_{tt}\left({t}_{0},{u}_{0}\right)]}^{-1/2}\right\}\left\{{\upsilon }_{0}^{-1}-{({u}_{0}G)}^{-1}\right\}/2\pi,$$

where

1. $${\tau }_{1}=sgn({\widehat{t}}_{0}){\left\{-2({K}_{1}\left({\widehat{t}}_{0}\right)-{\widehat{t}}_{0}\tau ) \right\}}^{1/2};$$
2. $${w}_{{u}_{0}}=sgn({t}_{0}){\left[-2\left\{K\left({t}_{0},{u}_{0}\right)-{K}_{2}\left({u}_{0}\right)-{t}_{0}\tau \right\}\right]}^{1/2};$$
3. $${\upsilon }_{0}=sgn({u}_{0}){\left[-2\left\{K\left({t}_{0},{u}_{0}\right)-{K}_{1}\left({\widehat{t}}_{0}\right)-\left({t}_{0}-{\widehat{t}}_{0}\right)\tau -{u}_{0}\upsilon \right\}\right]}^{1/2};$$
4. $$b=({w}_{{u}_{0}}-{\tau }_{1})/{\upsilon }_{0};$$
5. $$G={\left.{\left[{K}_{uu}\left(t,u\right)-{\left({K}_{tu}\left(t,u\right)\right)}^{2}/{K}_{tt}\left(t,u\right)\right]}^{1/2}\right|}_{\left({t}_{0},{u}_{0}\right)};$$
6. $${\rho }_{1}=-b/{(1+{b}^{2})}^{1/2};$$
7. $${\upsilon }_{1}={(\upsilon }_{0}-b{\tau }_{1})/{(1+{b}^{2})}^{1/2}.$$

Here $\Phi (.,.,\rho )$ is the standard bivariate normal distribution, $\rho$ is the correlation between the two components, and $\Phi (.)$ and $\phi \left(.\right)$ are the standard normal distribution and density functions.

To get, the value of the approximation in (9), some functions are required which are as follows

$${K}_{tt}\left(t,u\right)=\frac{{\partial }^{2}}{{\partial t}^{2}}K\left(t,u\right)=\sum_{i=1}^{n}\frac{{{U}_{1i}}^{2}{\text{exp}}\left(t{U}_{1i}+{uU}_{1i}\right)}{{\left[1+{\text{exp}}\left(t{U}_{1i}+{uU}_{1i}\right)\right]}^{2}} ,$$

$${K}_{uu}\left(t,u\right)=\frac{{\partial }^{2}}{{\partial u}^{2}}K\left(t,u\right)=\sum_{i=1}^{n}\frac{{{U}_{2i}}^{2}{\text{exp}}\left(t{U}_{1i}+{uU}_{2i}\right)}{{\left[1+{\text{exp}}\left(t{U}_{1i}+{uU}_{2i}\right)\right]}^{2}} ,$$

$${K}_{tu}\left(t,u\right)=\frac{{\partial }^{2}}{\partial t\partial u}K\left(t,u\right)=\sum_{i=1}^{n}\frac{{U}_{1i}{U}_{2i}{\text{exp}}\left(t{U}_{1i}+{uU}_{2i}\right)}{{\left[1+{\text{exp}}\left(t{U}_{1i}+{uU}_{2i}\right)\right]}^{2}} ,$$

$${K}_{1}\left(t\right)=\sum_{i=1}^{n}\mathrm{log}\left\{\frac{1}{2}+\frac{1}{2}{\text{exp}}\left(t{U}_{1i}\right)\right\}+t{c}_{1},$$

and

$${K}_{2}\left(u\right)=\sum_{i=1}^{n}\mathrm{log}\left\{\frac{1}{2}+\frac{1}{2}{\text{exp}}\left(u{U}_{2i}\right)\right\}+u{c}_{2}.$$

## Illustrative examples and simulation studies

Three published real data sets are considered in this part to demonstrate the efficiency of the saddlepoint and normal approximations. Inclusive Monte Carlo simulation studies are also carried out to evaluate the accuracy of the saddlepoint approach compared to that of the traditional asymptotic method.

### Examples

The precision of different approaches to approximate the exact p-value of bivariate sign tests may be illustrated using some numerical examples. As a result, three published real data sets are provided in order to compare the saddlepoint approximation and normal approximation methods. For Data Set 1, ten adult sons and their fathers participated in a study to assess eye refractions. Positive refractions indicated long-sightedness, while negative refractions showed near-sightedness. The sons were part of a large group collected in northern Finland for infants born in 1966. Data set 1 is presented in Table 1. More details can be found about this data set in Rantakallio^31^. We can indicate that several authors used the data presented in Table 1 to clarify some procedures for bivariate sign tests, for example, see Brown et al.^9^. Data set 2 is a simple study of twelve cotton textile workers who were researched by Merchant et al.^32^ to determine the effects of cotton dust exposure. Before and after each participant’s 6-h exposure to cotton dust, several factors were measured for each worker, including the change in closing volume and white blood cell count. Dietz^5^ used data set 2 to clarify the procedures of his bivariate sign test. This data set was included in Table 1 of Dietz^5^. Data set 3 is from Samawi et al.^33^. These data represent the bilirubin levels in jaundiced infants staying in the neonatal intensive care unit. Physicians are interested in jaundice because it may have a significant influence on hearing and neurological development and is a risk factor for death. It would be extremely beneficial to physicians if they could test the hypothesis that boys and females have the same median bilirubin level when weight groups are matched. The data was collected from five hospitals in Jordan and was limited to births in the first six months of 1997. Samawi et al.^33^ took fifteen pairs of male and female patients from the hospital records.

**Table 1:** Refraction values for ten sons with their father.

Son		Father		Deviation	
Right eye	Left eye	Right eye	Left eye	${x}_{1}$	${x}_{2}$
+ 0.50	+ 0.50	− 0.62	− 1.25	+ 1.12	+ 1.75
− 2.75	− 3.37	− 1.00	− 1.00	− 1.75	− 2.37
+ 0.25	0.00	+ 2.75	+ 2.75	− 2.50	− 2.75
− 0.50	− 0.25	− 2.00	− 1.50	+ 1.50	+ 1.25
+ 0.75	+ 0.50	+ 0.50	+ 0.63	+ 0.25	− 0.13
− 2.50	− 2.75	+ 0.50	+ 0.50	− 3.00	− 3.25
+ 0.50	+ 0.25	+ 2.00	+ 2.50	− 1.50	− 2.25
− 1.00	− 3.12	− 2.50	− 2.62	+ 1.50	− 0.50
− 3.37	− 2.37	− 1.75	− 1.75	− 1.62	− 0.62
+ 0.50	+ 0.50	+ 1.50	+ 2.25	− 1.00	− 1.75

Table 2 shows the mid p-values for the three data sets for the Blumen^2^ and Brown et al.^9^ bivariate sign tests. Furthermore, the asymptotic normal p-values and saddlepoint p-values are also displayed in Table 2. In the remainder of this article, we refer to the Blumen^2^ and Brown et al.^9^ tests by test 1 and test 2, respectively. The simulated mid p-value (Sim) is derived based on ${10}^{6}$ permutations of the indicators {${\beta }_{i}$} by computing the ratio of cases in which **B** exceeds ${{\varvec{B}}}_{0}$ plus half the ratio of cases in which **B** equals to ${{\varvec{B}}}_{0}$.

**Table 2:** Simulated, saddlepoint and normal p-values for the three data sets.

Data set	Approximation method	p-value of test 1	p-value of test 2
Set 1	Simulation	0.028526	0.140301
	Saddlepoint	0.025114	0.163803
	Normal	0.024715	0.191185
Set 2	Simulation	0.0335290	0.000122
	Saddlepoint	0.0336029	0.000092
	Normal	0.0348106	0.000262
Set 3	Simulation	0.004873	0.291819
	Saddlepoint	0.004813	0.291004
	Normal	0.005380	0.285886

In all three data sets, the saddle point approximation outperformed the normal approximation in terms of the simulated mid p-value precision.

### Monte Carlo simulation study

Monte Carlo simulation studies are used to show the accuracy of the saddlepoint approximation over a wide range of simulated data from different bivariate distributions and different sample sizes. 1000 bivariate data sets of sizes $n= 20,$ 30, 40, and 60 are generated from the bivariate exponential distribution, bivariate logistic distribution, bivariate normal distribution and bivariate Poisson distribution. For generating bivariate data from normal and Poisson distributions, three cases are taken into account for the correlation coefficient between the two variables: weak, moderate, and strong. While the data are generated from the bivariate exponential and logistic distributions assuming independence between the two variables. For the four distributions the following results “Sad.P.”, “E.Sad.”, and “E.Nor.” are presented in Tables 3, 4, 5 and 6, where “Sad.P.” is the proportion of the 1000 data sets for which the saddlepoint p-value is closer to the simulated exact mid p-value than the normal, “E.Sad.” is the average relative absolute error of the saddlepoint approximation, and “E.Nor.” is the average relative absolute error of the normal approximation. The estimated type I error and power of the considered tests at the 0.05 significance level are displayed in Tables 7 and 8, respectively.

**Table 3:** The results of the comparison between the saddlepoint method and the normal approximation method based on simulated data from bivariate normal distribution with correlation coefficient $\rho$.

Test	$n$	20			30			40			60		
	$\rho$	0.1	0.5	0.9	0.1	0.5	0.9	0.1	0.5	0.9	0.1	0.5	0.9
Test 1	Sad.P	90.3	90.6	95.7	86.9	89.1	92.1	85.9	86.1	87.9	78.9	77.6	83.1
	E.sad.	0.003	0.017	0.186	0.003	0.016	0.044	0.002	0.033	0.053	0.001	0.016	0.047
	E.Nor.	0.027	0.197	0.721	0.0125	0.114	0.498	0.010	0.144	0.258	0.003	0.055	0.137
Test 2	Sad.P	94.6	94.3	93.9	94.5	96.2	98.6	92.8	95.5	91.0	89.6	93.5	96.6
	E.sad.	0.009	0.012	0.599	0.009	0.023	0.008	0.013	0.019	0.812	0.019	0.017	0.003
	E.Nor.	0.230	0.578	0.603	0.172	0.835	0.188	0.156	0.512	0.848	0.135	0.399	0.115

**Table 4:** The results of the comparison between the saddlepoint method and the normal approximation method based on simulated data from bivariate logistic distribution.

Test	$n$	20	30	40	60
Test 1	Sad.P	93.7	92.4	89.4	90.7
	E.sad.	0.001	0.009	0.001	0.059
	E.Nor.	0.13	0.077	0.008	0.330
Test 2	Sad.P	93.7	96.3	99.2	92.5
	E.sad.	0.058	0.043	0.016	0.211
	E.Nor.	0.835	0.775	0.265	1.877

**Table 5:** The results of the comparison between the saddlepoint method and the normal approximation method based on simulated data from bivariate exponential distribution.

test	$n$	20	30	40	60
Test 1	Sad.P	92.7	92.4	89.9	81.1
	E.sad.	0.032	0.052	0.044	0.049
	E.Nor.	0.329	0.589	0.354	0.178
Test 2	Sad.P	90	95.2	98.5	91.2
	E.sad.	0.072	0.004	0.19	0.057
	E.Nor.	0.404	0.195	0.455	0.329

**Table 6:** The results of the comparison between the saddlepoint method and the normal approximation method based on simulated data from bivariate Poisson distribution with correlation coefficient $\rho$.

Test	$n$	20			30			40			60		
	$\rho$	0.1	0.5	0.9	0.1	0.5	0.9	0.1	0.5	0.9	0.1	0.5	0.9
Test 1	Sad.P	92.3	92.1	92.0	87.6	88.8	91.1	86.8	85.4	86.9	80.5	79.4	81.4
	E.sad.	0.002	0.002	0.022	0.002	0.002	0.004	0.002	0.002	0.004	0.001	0.002	0.009
	E.Nor.	0.0131	0.013	0.022	0.009	0.014	0.025	0.007	0.009	0.018	0.003	0.007	0.036
Test 2	Sad.P	84.5	92.0	95.0	88.4	95.4	96.6	91.0	95.5	95.7	92.8	96.1	96.0
	E.sad.	0.031	0.096	0.705	0.028	0.066	0.118	0.029	0.106	0.247	0.016	0.107	0.251
	E.Nor.	0.299	1.340	7.460	0.293	1.550	7.910	0.308	2.550	13.500	0.241	2.090	10.800

**Table 7:** Empirical type I error rates at 0.05 significance level.

Test	$n$	20	30	40	60
Test 1	Saddlepoint method	0.057	0.054	0.047	0.051
	Normal method	0.054	0.053	0.047	0.051
	Simulation method	0.057	0.053	0.047	0.051
Test 2	Saddlepoint method	0.068	0.054	0.046	0.061
	Normal method	0.068	0.055	0.042	0.064
	Simulation method	0.071	0.055	0.047	0.060

**Table 8:** Empirical power at 0.05 significance level.

Test	$n$	20	30	40	60
Test 1	Saddlepoint method	0.280	0.998	0.970	0.445
	Normal method	0.287	0.998	0.971	0.440
	Simulation method	0.284	0.998	0.972	0.450
Test 2	Saddlepoint method	0.639	0.776	0.954	0.662
	Normal method	0.649	0.785	0.954	0.667
	Simulation method	0.639	0.776	0.954	0.664

We notice from Tables 3, 4, 5 and 6 that the mean absolute error of the proposed approximation method is less than that of the normal approximation method in all the assumed cases. Moreover, we can note that the convergence percentage of suggested approximation to the simulated exact p-values was not in any case less than 77.6%, but in some cases, it reached approximately 99.2%. It is observed that with increasing sample sizes there is an improvement in the normal approximation, but the saddlepoint approximation is more accurate and is closer to the simulated exact p-value, especially when the sample sizes are small.

**Figure 1:**
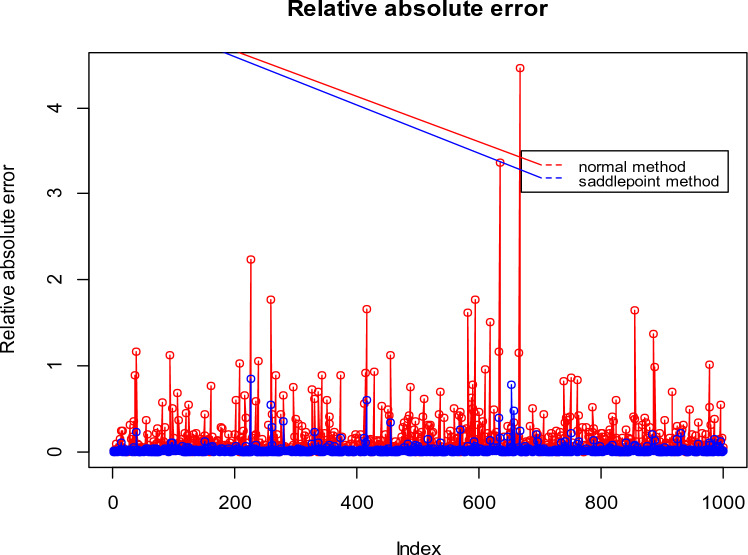
Relative absolute errors of saddlepoint approximation and normal approximation for Test 2 with sample size $n = 60$ generated from bivariate normal distribution.

**Figure 2:**
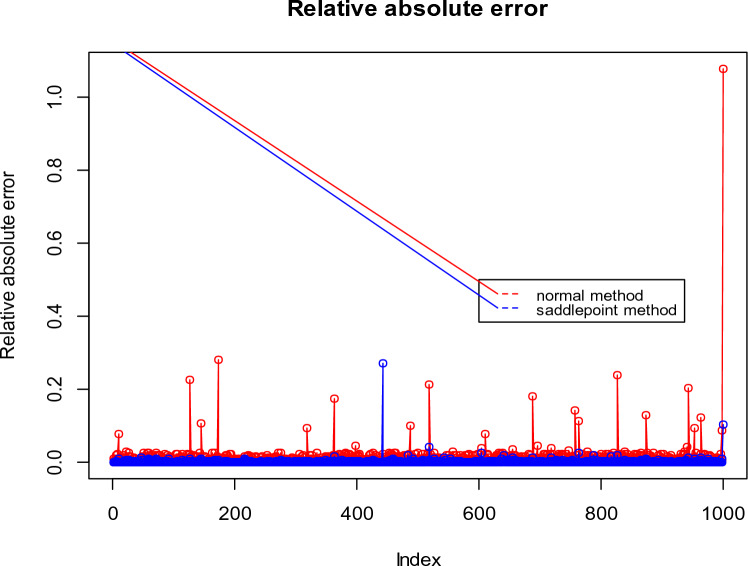
Relative absolute errors of saddlepoint approximation and normal approximation for the Test 1 with sample size $n = 20$ generated from bivariate Poisson distribution.

To facilitate a better understanding of the simulation results, the relative absolute errors of both the saddlepoint approximation and normal approximation for two cases of the simulation study are displayed in Figs. 1 and 2.

## Concluding

Bivariate data analysis is becoming more and more important in many areas. Especially in the medical field, more than one variable such as tumor incidence and tumor size, blood pressure and pulse, weight and fat level, weight change and depression level are often studied. Leveraging Wang’s bivariate saddlepoint approximation technique, the exact p-values of a class of bivariate sign tests are approximated with high precision compared to normal approximation method. This high accuracy has been verified by analyzing three examples of real data and performing simulation study.

## Data Availability

The datasets used and/or analysed during the current study available from the corresponding author on reasonable request.
