# Author Correction: A new approach for approximating the p-value of a class of bivariate sign tests

**DOI:** 10.1038/s41598-023-49383-9

**Published:** 2023-12-19

**Authors:** Ibrahim A. A. Shanan, Ehab F. Abd-Elfattah, Abd El-Raheem M. Abd El-Raheem

**Affiliations:** 1 Department of Mathematics, Faculty of Education, Ain Shams University, Cairo, Egypt; 2 Department of Information Technologies, Management Technical College, Southern Technical University, Basra, Iraq

Correction to: *Scientific Reports* 10.1038/s41598-023-45975-7, published online 05 November 2023

The original version of this Article contained an error in the order of the author names, which was incorrectly given as Ibrahim A. A. Shanan, Abd El-Raheem M. Abd El-Raheem, Ehab F. Abd-Elfattah.

In addition, the original version of this Article contained an error in the Funding section.

"Open access funding provided by The Science, Technology & Innovation Funding Authority (STDF) in cooperation with The Egyptian Knowledge Bank (EKB). The funding was also provided by Science and Technology Development Fund."

now reads:

“Authors state no funding involved."

The original Article has been corrected.

